# Supplementary material for: SND3 is the membrane insertase within a distinct SEC61 translocon complex
Source: Nat Commun. 2025 Oct 29;16:9566. doi: 10.1038/s41467-025-65357-z (PMC12572126; doi:10.1038/s41467-025-65357-z)
Supplement: Supplementary file 1 — Supplementary Information [file 41467_2025_65357_MOESM1_ESM.pdf]

Supplementary Figures

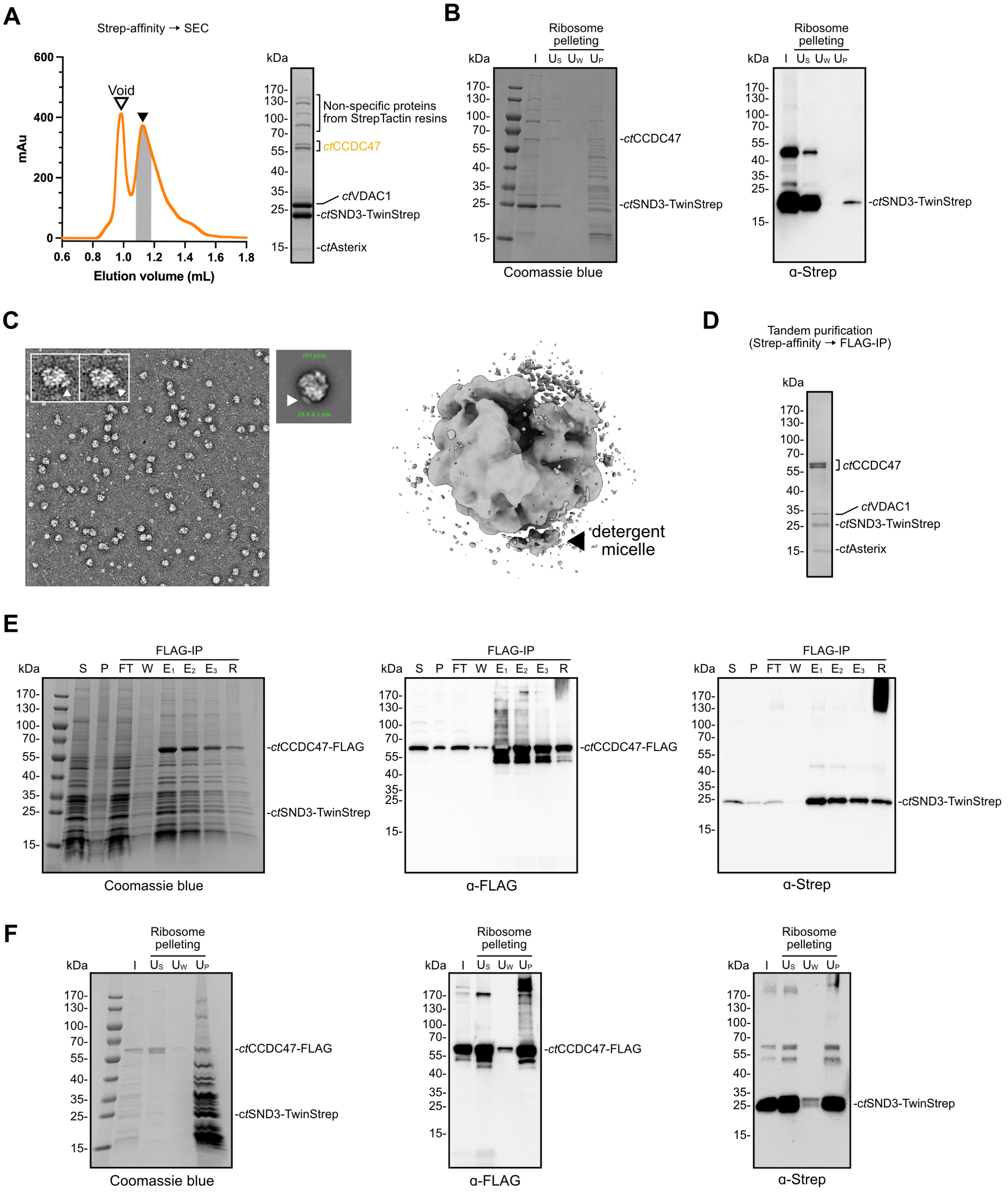

Supplementary Fig. 1. Purification strategies for isolating ribosome-bound SND3-associated complexes.

(**A**) (Left) SEC profile of StrepTactin affinity-purified *ct*SND3-TwinStrep complexes from the *C. thermophilum* *ct*SND3-TwinStrep strain. (Right) The single fraction highlighted in grey was analysed by SDS-PAGE with Coomassie blue staining. The gel bands for *ct*SND3, *ct*CCDC47 and *ct*VDAC1 were identified by MS analysis. The band for *ct*Asterix is inferred from MS analysis of the gel in **D**. (**B**) Additional ultracentrifugation after StrepTactin affinity purification of *ct*SND3-TwinStrep showed a small proportion of SND3 in the ribosome-containing pellet (U_P) when separated by SDS-PAGE. Samples from the affinity-purified *ct*SND3 input (I), ribosome-free supernatant (U_S) and pellet wash (U_W) are also analysed by Coomassie blue staining (left) or western blotting with monoclonal anti-Strep antibody (right). (**C**) Negative stain EM analysis of the ribosome-containing fraction in **B** showing a representative micrograph and exemplary particles (left), an example 2D class average (middle) and the resulting low-resolution map (right). Arrows in each image indicate the associated density at the ribosome tunnel exit. (**D**) SDS-PAGE analysis and Coomassie blue staining of the complex obtained after tandem StrepTactin-affinity purification and FLAG-IP from the *C. thermophilum* *ct*SND3-TwinStrep/*ct*CCDC47-FLAG strain. The labelled gel bands were identified by MS analysis. (**E-F**) Samples taken during (**E**) FLAG-IP of *ct*CCDC47-FLAG and (**F**) further ribosome pelleting were separated by SDS-PAGE and visualised by Coomassie blue staining and western blotting with anti-FLAG or anti-Strep antibodies as indicated. Aside from ribosome pelleting samples defined in **B**, samples were taken from the soluble fraction after detergent solubilisation (S), the insoluble fraction after detergent solubilisation (P), the unbound fraction from the FLAG-IP (FT), the wash fraction from the FLAG-IP (W), the three separate elution fractions from the FLAG-IP (E_{1-3}) and the anti-FLAG M2 affinity gel after elution (R). All purifications were performed at least twice and uncropped images are provided in the Source Data file.

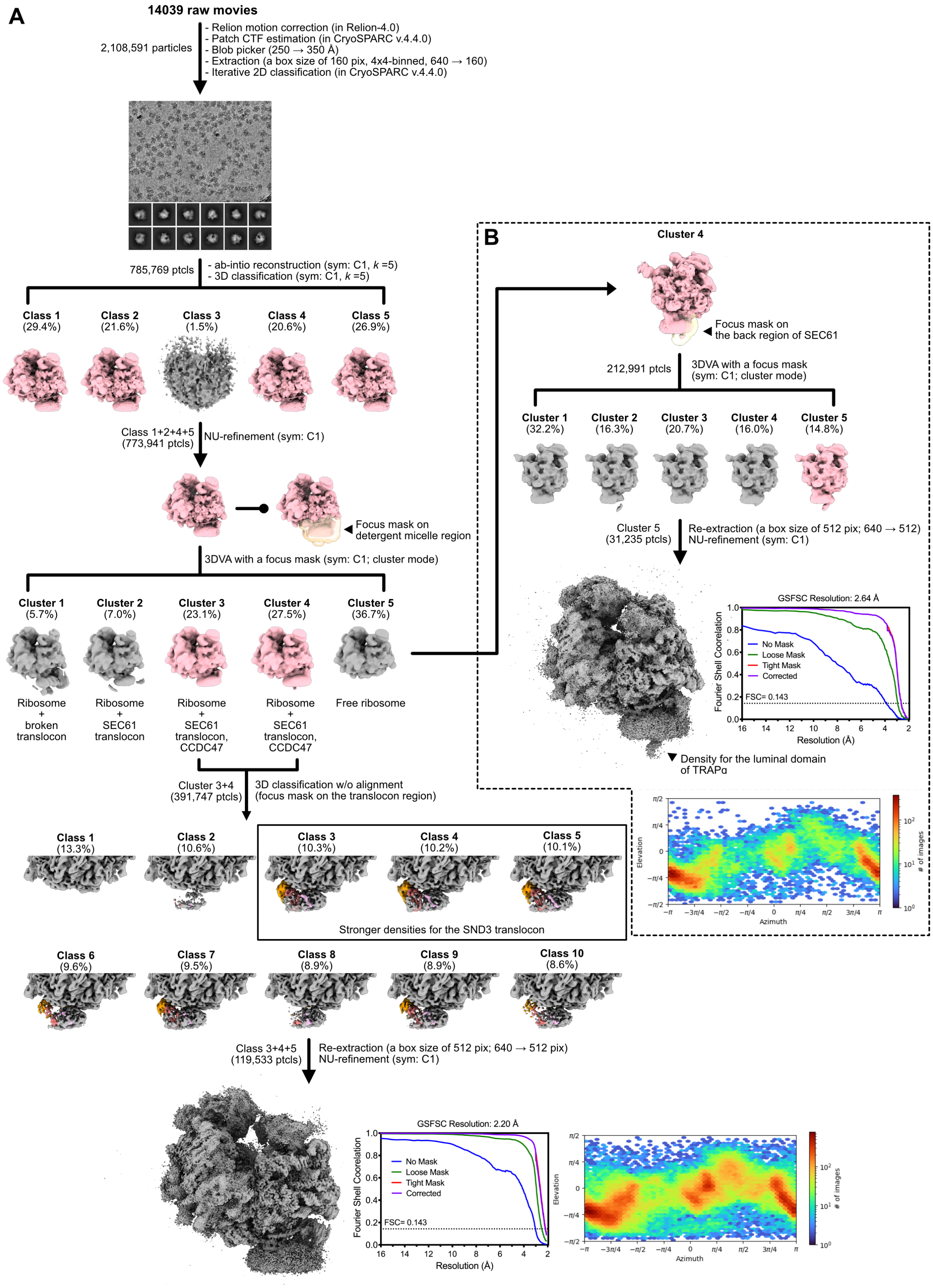
A
14039 raw movies
2,108,591 particles
- Relion motion correction (in Relion-4.0)
- Patch CTF estimation (in CryoSPARC v.4.4.0)
- Blob picker (250 → 350 Å)
- Extraction (a box size of 160 pix, 4x4-binned, 640 → 160)
- Iterative 2D classification (in CryoSPARC v.4.4.0)
785,769 ptcls
- ab-intio reconstruction (sym: C1, k =5)
- 3D classification (sym: C1, k =5)
Class 1 (29.4%)
Class 2 (21.6%)
Class 3 (1.5%)
Class 4 (20.6%)
Class 5 (26.9%)
Class 1+2+4+5 (773,941 ptcls)
NU-refinement (sym: C1)
Focus mask on detergent micelle region
3DVA with a focus mask (sym: C1; cluster mode)
Cluster 1 (5.7%)
Cluster 2 (7.0%)
Cluster 3 (23.1%)
Cluster 4 (27.5%)
Cluster 5 (36.7%)
Ribosome + broken translocon
Ribosome + SEC61 translocon
Ribosome + SEC61 translocon, CCDC47
Ribosome + SEC61 translocon, CCDC47
Free ribosome
Cluster 3+4 (391,747 ptcls)
3D classification w/o alignment
(focus mask on the translocon region)
Class 1 (13.3%)
Class 2 (10.6%)
Class 3 (10.3%)
Class 4 (10.2%)
Class 5 (10.1%)
Stronger densities for the SND3 translocon
Class 6 (9.6%)
Class 7 (9.5%)
Class 8 (8.9%)
Class 9 (8.9%)
Class 10 (8.6%)
Class 3+4+5 (119,533 ptcls)
Re-extraction (a box size of 512 pix; 640 → 512 pix)
NU-refinement (sym: C1)
GSFSC Resolution: 2.20 Å
Fourier Shell Coorelation
No Mask
Loose Mask
Tight Mask
Corrected
FSC= 0.143
Resolution (Å)
Elevation
Azimuth
# of images
B
Cluster 4
Focus mask on the back region of SEC61
212,991 ptcls
3DVA with a focus mask (sym: C1; cluster mode)
Cluster 1 (32.2%)
Cluster 2 (16.3%)
Cluster 3 (20.7%)
Cluster 4 (16.0%)
Cluster 5 (14.8%)
Cluster 5 (31,235 ptcls)
Re-extraction (a box size of 512 pix; 640 → 512)
NU-refinement (sym: C1)
GSFSC Resolution: 2.64 Å
Fourier Shell Coorelation
No Mask
Loose Mask
Tight Mask
Corrected
FSC= 0.143
Resolution (Å)
Density for the luminal domain of TRAPα
Elevation
Azimuth
# of images

Supplementary Fig. 2. Workflow for single-particle cryo-EM data processing of the ribosome-bound SND3 translocon.

(**A**) Overview of the cryo-EM data processing of the ribosome-bound SND3 translocon. The micrographs that meet the selection criterion of CTF_fit_resolution < 4 Å were used for particle selection, iterative rounds of 2D classification, 3D classification, and non-uniform (NU) refinement. The resulting cryo-EM map was used for 3D variability analysis (3DVA) with a mask focusing on the detergent micelle region. Two selected 3DVA classes were pooled and further classified by a round of focused 3D classification without alignment. The resulting three classes were selected and used to generate the final cryo-EM map of the ribosome-bound SND3 translocon at an overall resolution of 2.20 Å. (**B**) The 3DVA cluster 4 was independently selected for further processing with a focused mask on the back region of SEC61, resulting in a second cryo-EM map with improved density for the luminal domain of *ct*TRAPα. Gold standard FSC curves and the angular distribution of particles used are shown next to the corresponding final reconstructions.

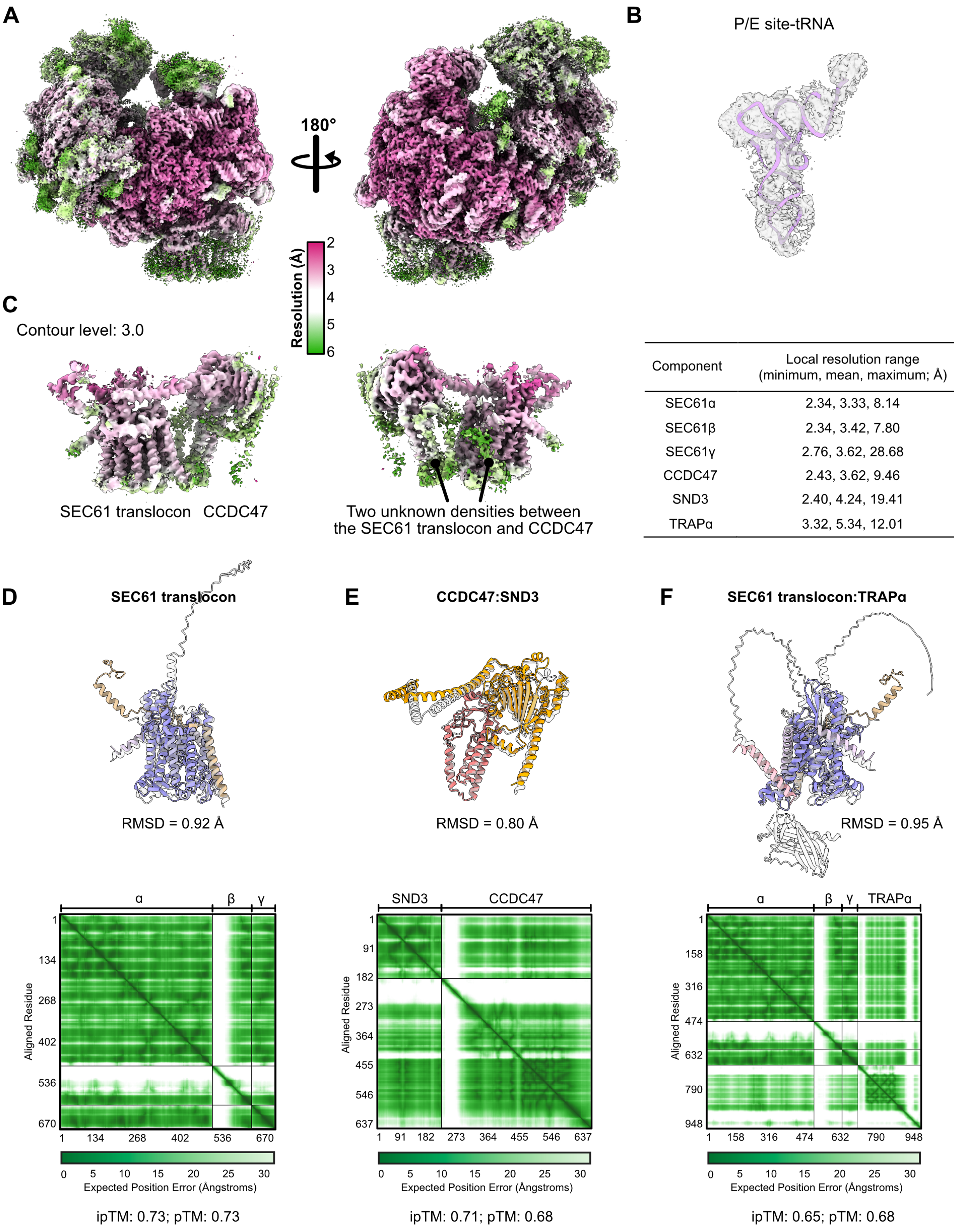

Component	Local resolution range (minimum, mean, maximum; Å)
SEC61α	2.34, 3.33, 8.14
SEC61β	2.34, 3.42, 7.80
SEC61γ	2.76, 3.62, 28.68
CCDC47	2.43, 3.62, 9.46
SND3	2.40, 4.24, 19.41
TRAPα	3.32, 5.34, 12.01

Supplementary Fig. 3. Local resolution estimates and model building for the ribosome-bound SND3 translocon.

(**A**) Local resolution estimation of the overall cryo-EM structure of the ribosome-bound SND3 translocon. (**B**) Superimposition of the final cryo-EM map with the structure of the pe/E-site tRNA from PDB 7OLD. (**C**) (Left) Zoom-in of the detergent micelle region for the views shown in **A**. The contour level of the cryo-EM map was set to 3.0 in UCSF-ChimeraX[59]. The schematics reveal two clear densities for the *ct*SEC61 translocon and *ct*CCDC47 and two unknown densities between the *ct*SEC61 translocon and *ct*CCDC47. (Right) Local resolution ranges of the individual components derived from UCSF-ChimeraX. (**D-E**) Comparison of the AF3[21] models (grey) with the final models (coloured as in Fig. 1C) for the (**D**) *ct*SEC61 translocon, (**E**) *ct*CCDC47/SND3 subcomplex and (**F**) *ct*SEC61 translocon/TRAPα subcomplex, showing the respective RMSD for each superimposition. Confidence metrics for the AF3 models are shown, including a plot of the predicted alignment error (PAE) and scores for predicted template modeling (pTM) and the interface predicted template modeling (ipTM).

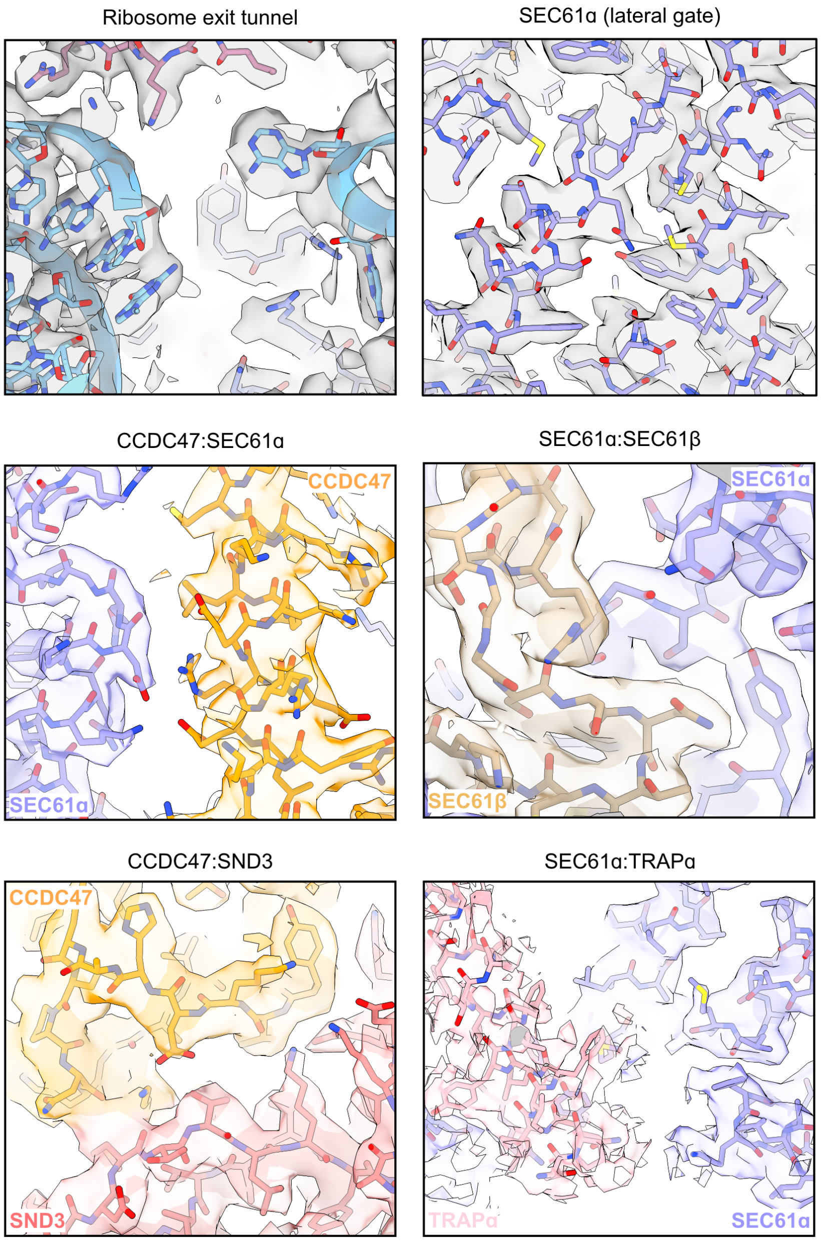

Supplementary Fig. 4. Density quality within the ribosome-bound SND3 translocon cryo-EM map.

Representative density and its fit to the corresponding structural model are shown for the indicated regions of the ribosome-associated SND3 translocon.

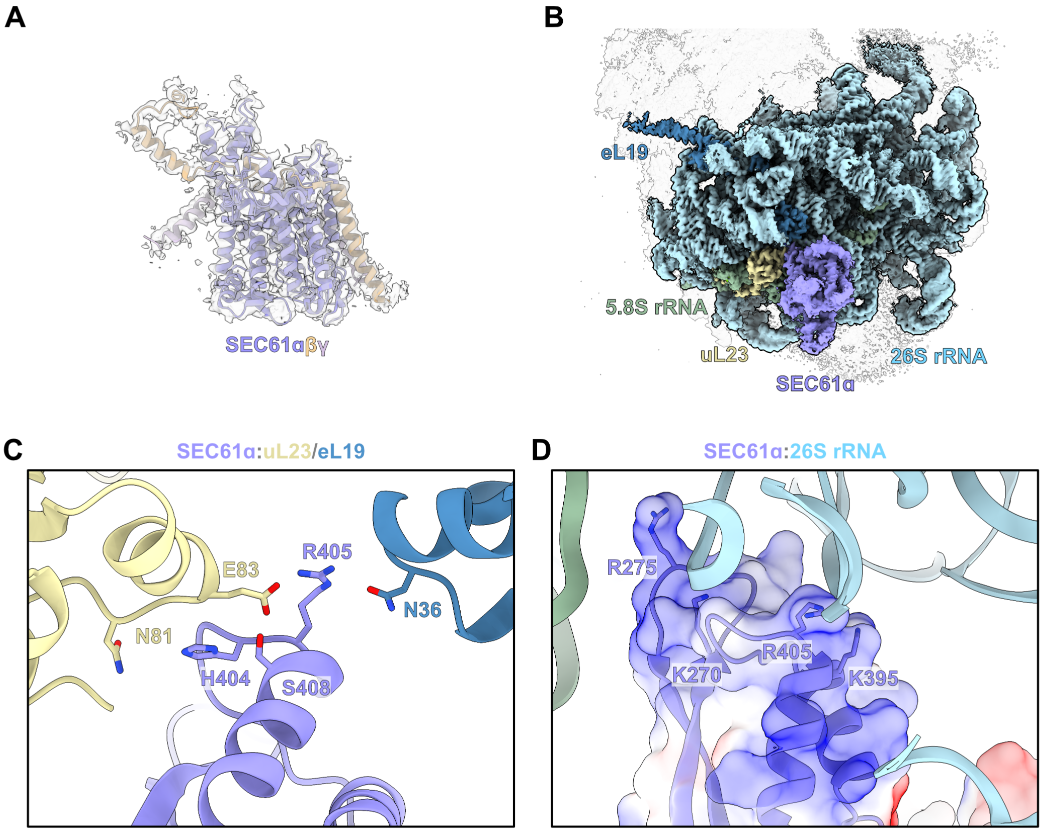

Supplementary Fig. 5. Interactions of SEC61α with the 60S ribosomal subunit.

(**A**) Final model for the *ct*SEC61 translocon within the cryo-EM density. (**B**) Overview of the interactions between *ct*SEC61 and the 60S ribosomal subunit. Detailed views of the interactions of the cytosolic loops within the C-terminal half of *ct*SEC61 with (**C**) uL23 and eL19, and (**D**) 26S rRNA are shown.

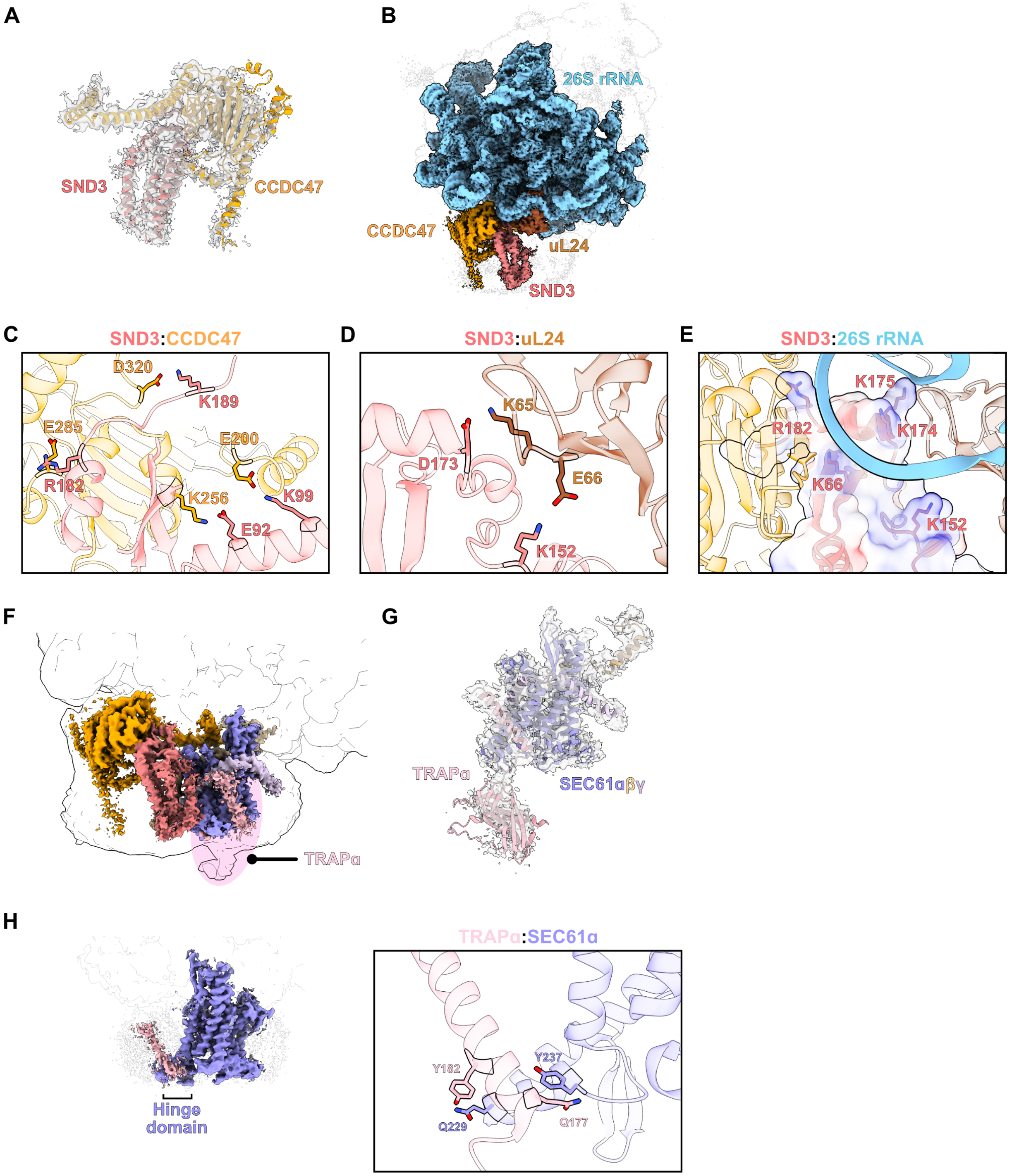

Supplementary Fig. 6. Interactions of SND3 and TRAPα within the SND3 translocon.

(**A**) Final model for the *ct*CCDC47/SND3 complex within the cryo-EM density. (**B**) Overview of the interaction partners of *ct*SND3 within the SND3 translocon complex. Detailed views of the interactions of *ct*SND3 with (**C**) *ct*CCDC47, (**D**) uL24, and (**E**) 26S rRNA are shown. (**F**)

Superimposition of the final cryo-EM map and the corresponding binned map (4x binning) at low contour levels. This schematic reveals the second unknown density comprises a TMD and an ER-luminal domain, which resembles the structure of TRAPα. (**G**) Final model for the *ct*SEC61 translocon/TRAPα complex within the improved cryo-EM map shown in Supplementary Fig. 2B. (**H**) Views of the interaction between the TMD of *ct*TRAPα and the hinge domain of *ct*SEC61α in the cryo-EM reconstruction (left) and model (right).

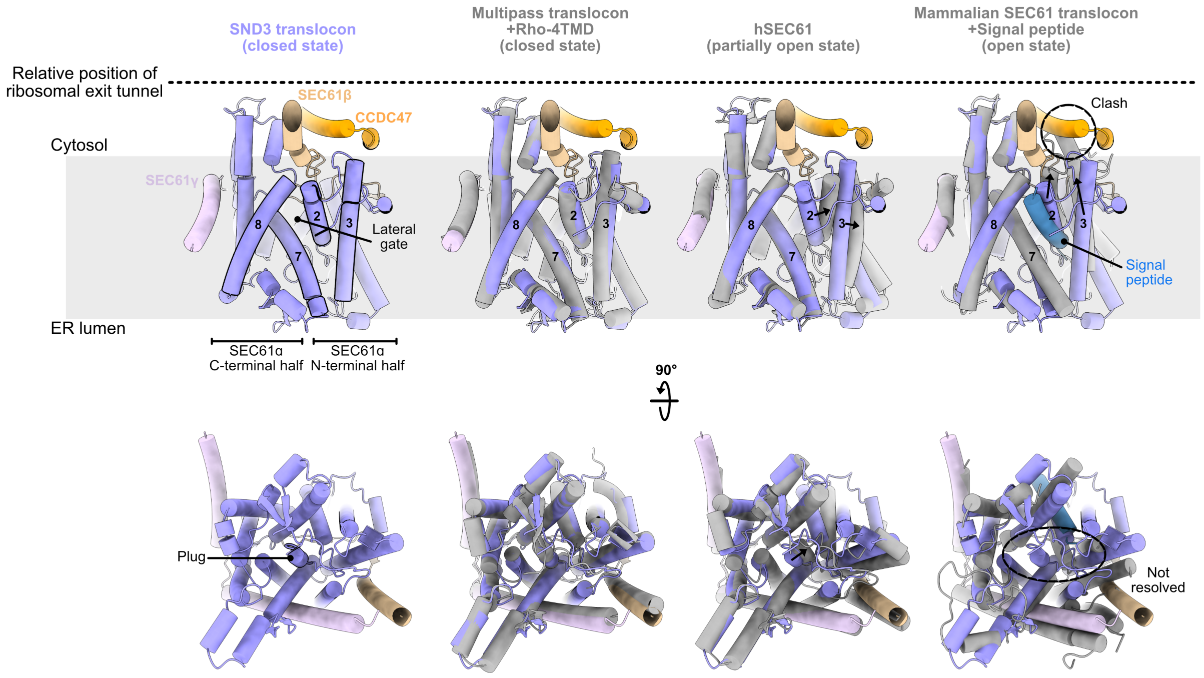

Supplementary Fig. 7. The SEC61α channel adopts a closed conformation in the SND3 translocon.

Side-by-side comparison between *ct*SEC61α in the SND3 translocon (purple) and representative structures of the channel in different states (grey). The C-terminal half of *ct*SEC61α is superimposed with structures for the closed (PDB 7TUT; RMSD 1.06 Å over 241 atoms), partially open (PDB 8DNV; RMSD 1.13 Å over 210 atoms) and open (PDB 3JC2; RMSD 1.01 Å over 132 atoms) SEC61α channel structures. (Top) View from the membrane plane. Arrows show the different position for TMD2 and TMD3 in the partially open and open channels, and the circle highlights a clash between *ct*CCDC47 (orange) and the cytosolic end of TMD3 in the open channel. (Bottom) View from the ER lumen. The arrow shows the movement of the plug helix in the partially open channel and the circle indicates that the plug helix is not resolved in the open channel.

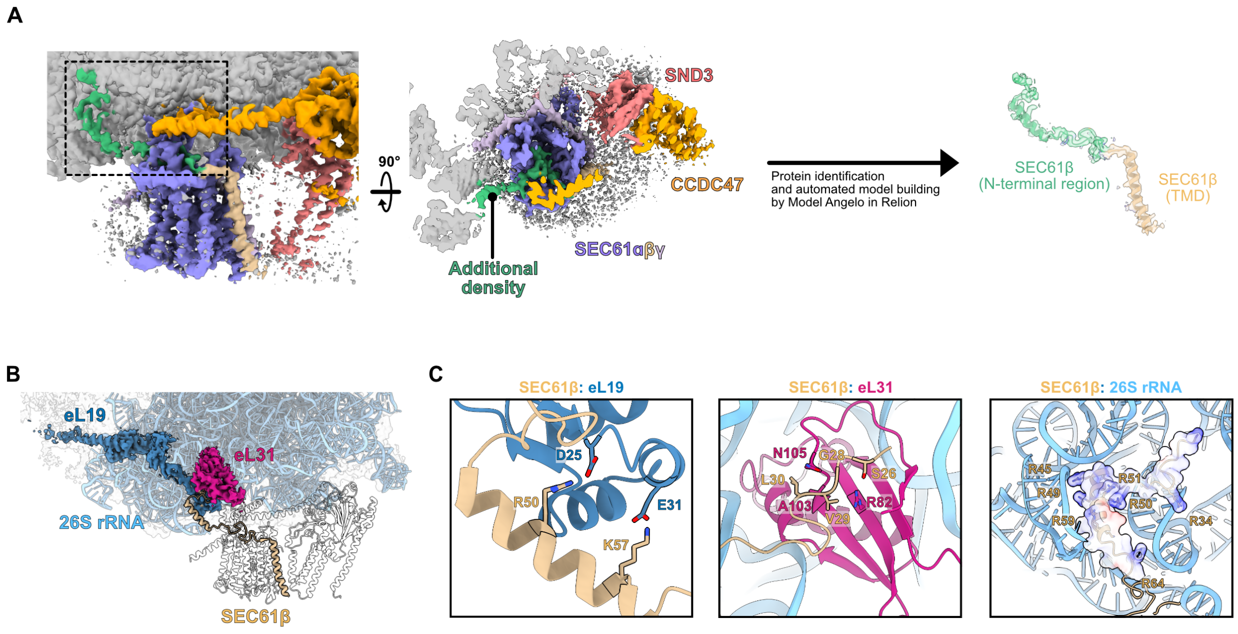

Supplementary Fig. 8. Structural characterisation of the N-terminal region of SEC61β.

(**A**) Additional density (green) is found attached to the ribosome and extends to the entry of the *ct*SEC61 translocon. Protein identification as the N-terminal region of *ct*SEC61β and automatic model building in the density were performed using Model Angelo[27]. (**B**) Overview of *ct*SEC61β interactions with the ribosome. (**C**) Detailed views of the interfaces between the *ct*SEC61β ribosome binding domain and eL19, eL31 and 26S rRNA.

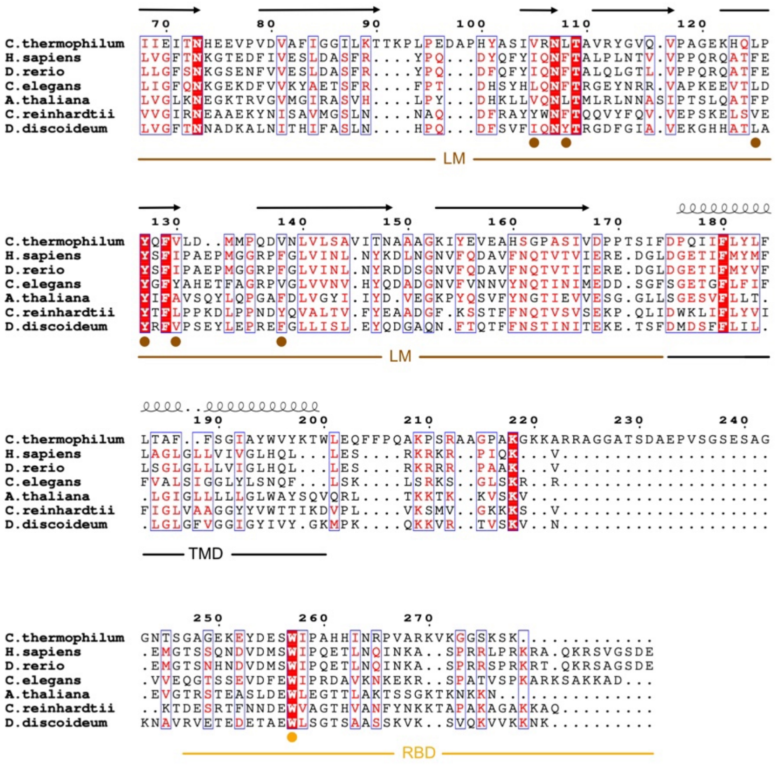

Supplementary Fig. 9. TRAPα sequence conservation in eukaryotes.

Sequence alignment of TRAPα homologues generated using Clustal Omega[91] and visualised using Espript 3.0[92]. Secondary structure elements from the AF3 model of *ct*TRAPα are shown above the sequence for the luminal domain (LM). Circles below the alignment highlight conserved residues shown to be functionally important in the LM[23] (brown) and for the interaction between the TRAPα ribosome binding domain (RBD) and the ribosome (orange).

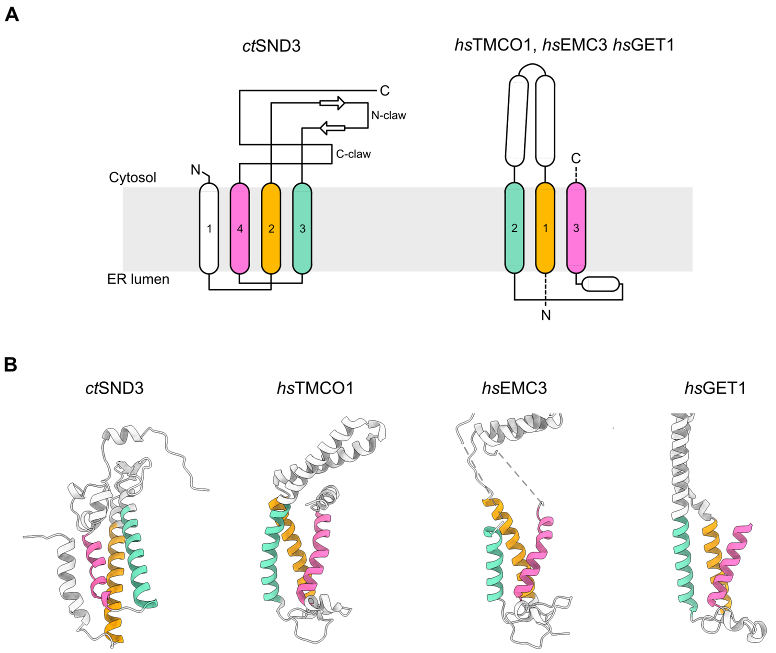

Supplementary Fig. 10. *ct*SND3 does not have the fold of an Oxa1 superfamily insertase.

(**A**) Schematic representation of the topology of *ct*SND3 and human (*hs*) Oxa1 superfamily membrane insertases showing a swapped TMD organisation and different secondary structure of the extramembranous loops relative to *ct*SND3. The three TMDs contributing to the membrane-embedded hydrophilic groove are coloured in sequence order. (**B**) Side-by-side comparison of the structures of *ct*SND3 and human Oxa1 superfamily membrane insertases coloured as in **A**. *hs*EMC3 (PDB 6WW7) was superimposed with *hs*TMCO1 (PDB 7TUT; RMSD 1.40 Å over 30 atoms) and *hs*GET1 (PDB 8CR1; RMSD 1.17 Å over 31 atoms).

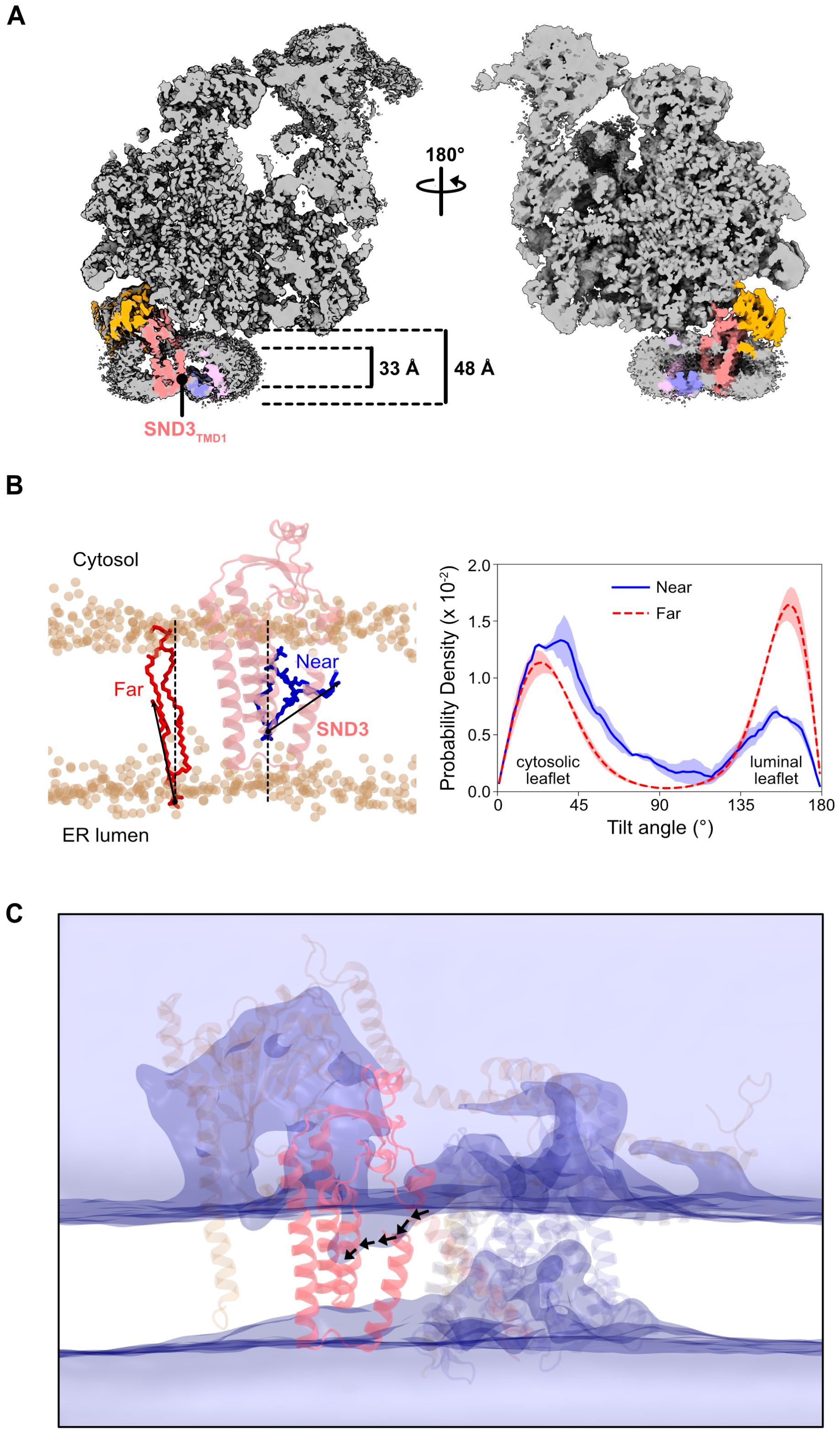
A
180°
33 Å
48 Å
SND3$_{TMD1}$
B
Cytosol
Near
Far
SND3
ER lumen
Probability Density (x 10^{-2})
2.0
1.5
1.0
0.5
0.0
Near
Far
cytosolic leaflet
luminal leaflet
0
45
90
135
180
Tilt angle (°)
C

Supplementary Fig. 11. ***ct*****SND3 causes local membrane thinning.**

(**A**) Slices of the cryo-EM map of the SND3 translocon through the membrane normal demonstrate local detergent micelle thinning occurring in the vicinity of *ct*SND3 TMD1. The density corresponding to each SND3 translocon component is coloured as in Fig. 1B. (**B**) (Left) An atomistic simulation snapshot showing the phospholipid phosphorus (P) atoms and *ct*SND3, where phospholipids are categorised as 'near' (blue; P atom within 5 Å of any heavy atom of *ct*SND3) or 'far' (red; P atom more than 10 Å away from any heavy atom of *ct*SND3). The dashed line shows the membrane normal and the solid arrow shows the hydrophobic tail vector of the phospholipid. (Right) The probability density of phospholipid tail distortion near (blue) and far (red) from *ct*SND3 obtained from atomistic MD simulations. The tilt was determined by measuring the angle of the hydrophobic tail vector of the phospholipid relative to the z-axis (membrane normal) starting from 0° (cytosolic leaflet) or from 180° (luminal leaflet). The solid line shows the mean distribution from three simulation replicates and the shaded region represents the standard error over these replicates. These distributions reveal distortions in lipid orientation near *ct*SND3 particularly in the cytosolic membrane leaflet. Source data are provided in the Source Data file. (**C**) Snapshot of an atomistic MD simulation showing the time-averaged density of water above and below the membrane as a transparent blue isosurface. The structure of the SND3 translocon is shown in cartoon representation, in which *ct*SND3 is opaque and other components are transparent. The arrows indicate a pathway for water molecules from the cytosol to the hydrophilic groove of *ct*SND3.

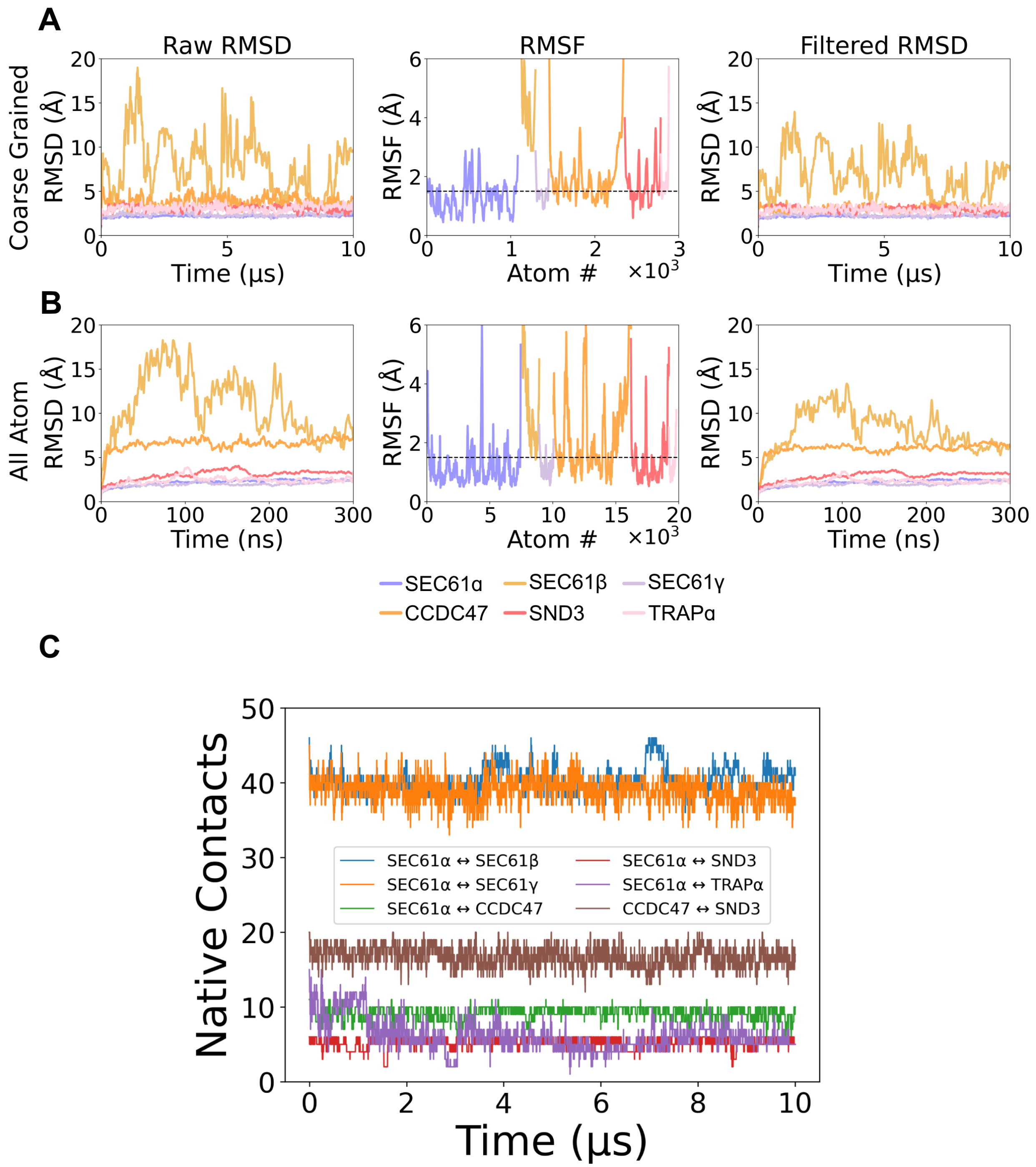

Supplementary Fig. 12. Stability of the protein subunits and their interfaces in the SND3 translocon.

Root mean square deviations (RMSD) and root mean square fluctuations (RMSF) calculated from (**A**) coarse-grained simulations and (**B**) all-atom simulations of the SND3 translocon. The raw RMSD of the six protein subunits calculated from the simulation trajectories and the RMSF values

of the heavy atoms of the subunits are shown in the left and middle panels respectively. Filtered RMSD shown in the right panel represents the RMSD values of the proteins fitted to the atoms with RMSF < 1.5 Å (dashed black line indicated in the middle panel). The RMSF cut-off for SEC61β in **A** was taken as 3.5 Å because of its overall high RMSF. (**C**) Time evolution of the number of native contacts between pairs of subunits in the translocon complex in the coarse-grained simulation. A native contact was defined as a distance < 4.5 Å between any two heavy atoms of a subunit pair in the experimental structure. Each contact was considered to remain formed until the minimum distance between the atom-pair crossed 7 Å (to filter out transient openings) beyond which the contact was considered broken until it again reached < 4.5 Å. Protein pairs with less than five native contacts were not included. Source data are provided in the Source Data file.

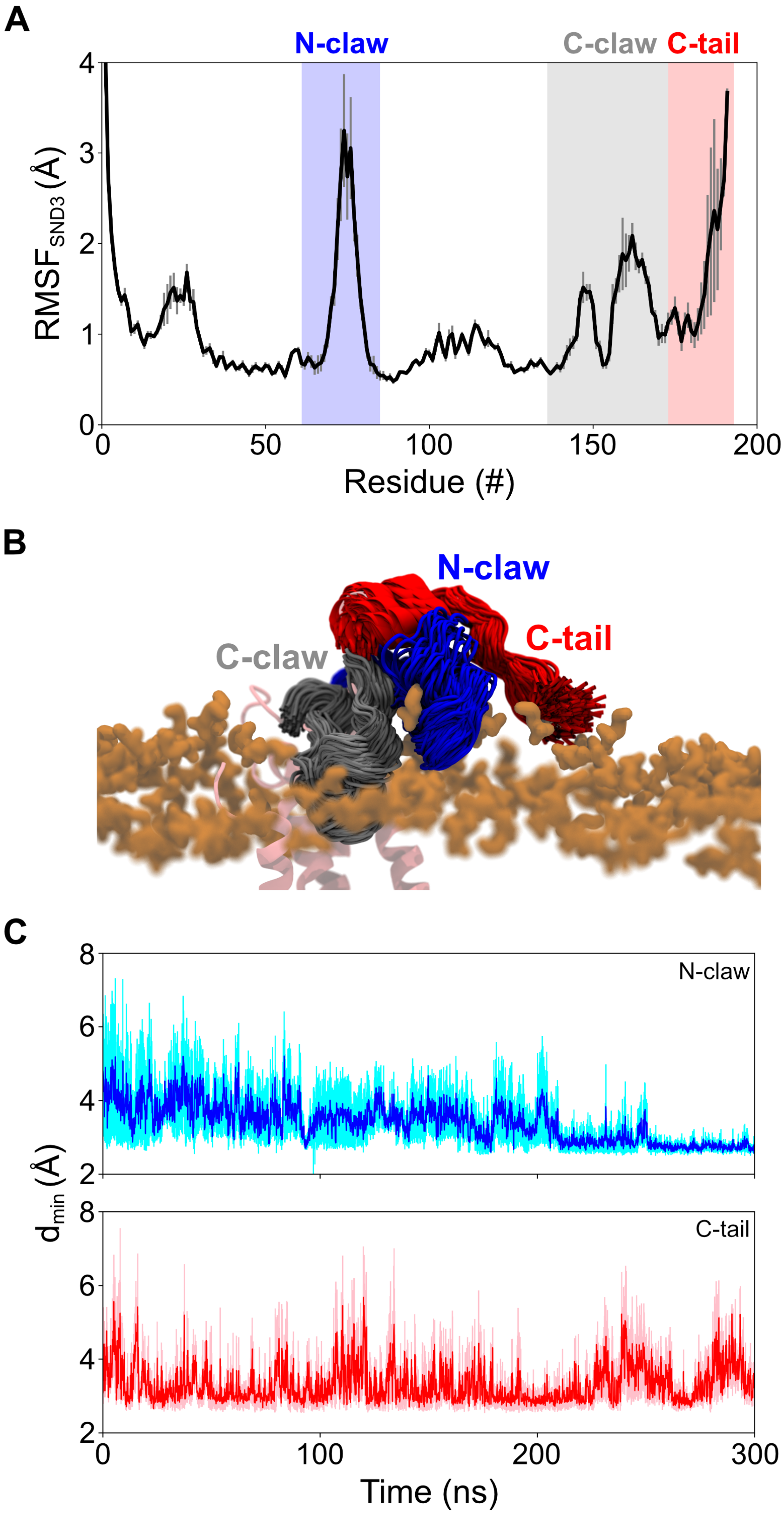
A
N-claw
C-claw
C-tail
RMSF$_{SND3}$ (Å)
Residue (#)
B
N-claw
C-tail
C-claw
C
N-claw
C-tail
d_{min} (Å)
Time (ns)

Supplementary Fig. 13. Dynamics and membrane association of the *ct*SND3 cytosolic domains.

(**A**) Root mean square fluctuations (RMSF) of *ct*SND3 C_α atoms averaged over three atomistic MD simulation replicates. The error bars (grey) represent the standard error over the three replicates. The N-claw, C-claw and C-tail of *ct*SND3 are highlighted by the blue, grey and red rectangles, respectively. (**B**) The N-claw (blue; residues K60 to V86), C-claw (grey; residues S135 to A172) and C-tail (red; residues D173-E191) are shown in cartoon representation as superimpositions of snapshots at 300 equally spaced time frames in 300 ns of atomistic MD simulation. The remaining *ct*SND3 backbone is shown as cartoon and the lipid headgroups are shown as brown surfaces. (**C**) Time traces of the minimum distances between the heavy atoms of the N-claw or C-tail and the lipid headgroups. Only the membrane proximal regions from **B** for the N-claw (residues A71-E78) and C-tail (residues G184-E191) are used for the analysis. The values plotted here are means over three independent simulations. The error bars represent the standard error over the three replicates. Source data for the graphs are provided in the Source Data file.

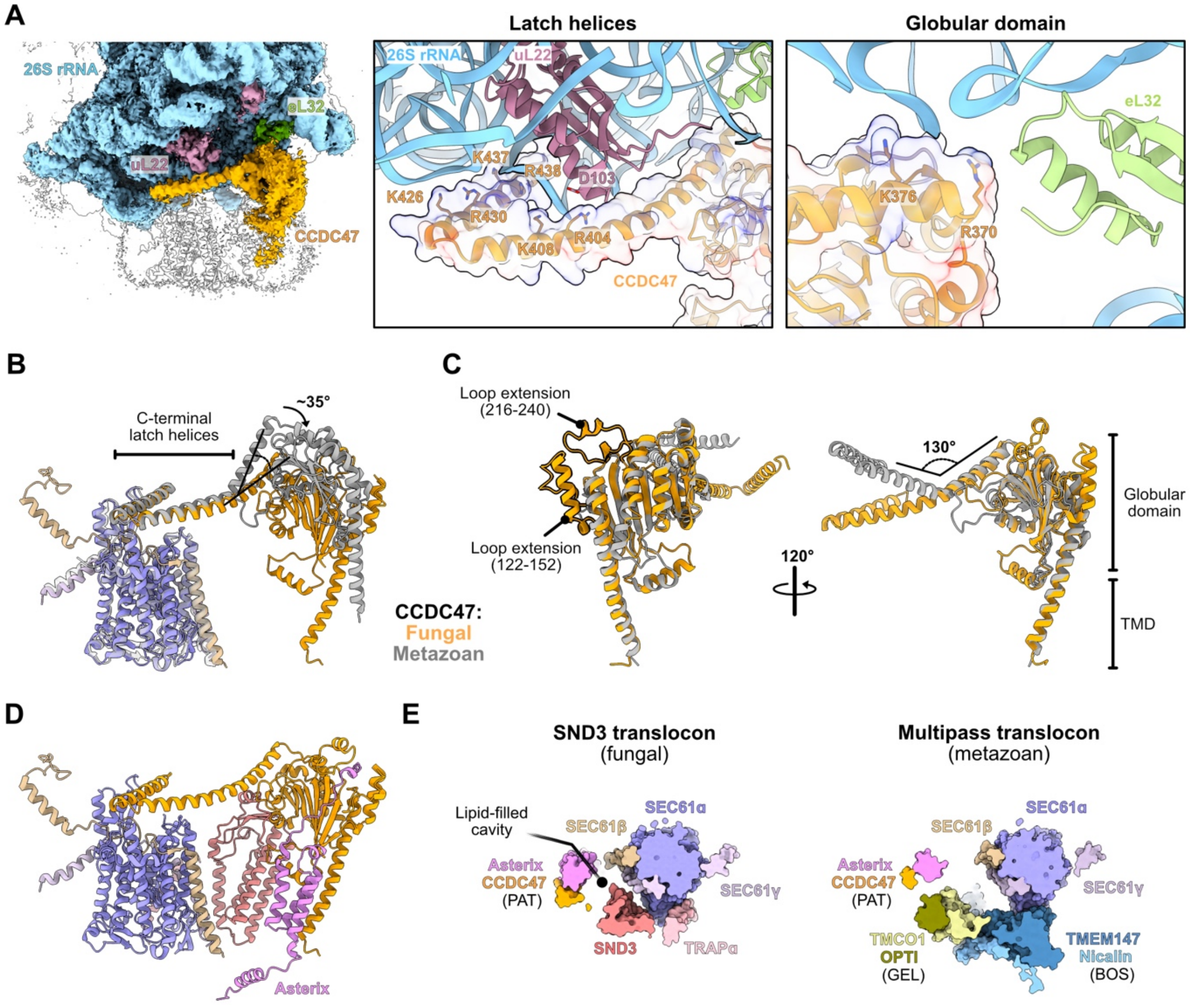

Supplementary Fig. 14. Comparison of the PAT complex between the fungal and metazoan MPT.

(**A**) Overview of *ct*CCDC47 interactions with the ribosome (left), showing detailed views of the interfaces with uL22, eL32 and 26S rRNA (right). The interface with the *ct*CCDC47 latch helices is conserved with the metazoan MPT, whilst the interface with the globular domain is not. (**B**) Structural comparison of the SND3 translocon (colour) and metazoan MPT (grey; PDB 7TUT), superimposed as in Fig. 4. The latch helices of CCDC47 are positioned identically relative to the SEC61 translocon, but the globular domains are titled by ~35° with respect to each other. (**C**) Superimposition of the TMD and globular domain of fungal (orange) and metazoan CCDC47 (grey; RMSD 1.30 Å over 170 atoms) indicates a largely similar fold aside from the two indicated helical loop extensions in *ct*CCDC47. The connection to the latch helices is kinked by 130° in metazoan CCDC47 resulting in the repositioning shown in **B**. (**D**) Model for the interaction of

*ct*Asterix with the SND3 translocon shown in the same view as Fig. 1C. The position of *ct*Asterix was derived from an AF3[21] model for the *ct*Asterix/CCDC47/SND3 ternary complex that had been superimposed with *ct*CCDC47 in our structure (RMSD 0.80 Å over 294 atoms). (**E**) Comparison of the model in **D** with the metazoan MPT, superimposed and represented as in Fig. 4A.

		Fungi	Metazoa	Viridiplantae	Alveolata	Stramenopiles	Euglenozoa	Amoebozoa	
SND3		2107	3	14	16	137	29	9	fungal MPT
TMCO1	GEL	79	1521	425	70	6	0	15	
OPTI		75	1426	479	46	6	0	9	
TMEM147		0	1423	411	47	2	0	8	metazoan MPT
Nicalin	BOS	0	1830	444	45	8	27	0	
NOMO		0	1690	423	5	5	0	11	
CCDC47	PAT	1890	1655	525	68	33	1	15	
Asterix		1435	1378	377	9	1	1	8	
SEC61α		2393	2269	567	116	97	36	18	conserved components
SEC61β		2334	1097	1003	87	58	4	5	
SEC61γ		1781	2864	27	70	73	24	15	
TRAPα		1806	1783	616	1	9	29	10	

Supplementary Fig. 15. Mutually exclusive conservation of the fungal and metazoan MPT.

Homologues for the fungal-specific, metazoan-specific and conserved MPT components were identified in the given eukaryotic taxa using OrthoDB v12.0[90]. Each column is coloured on an independent relative scale of lowest (white) to highest (dark grey) number of homologues.

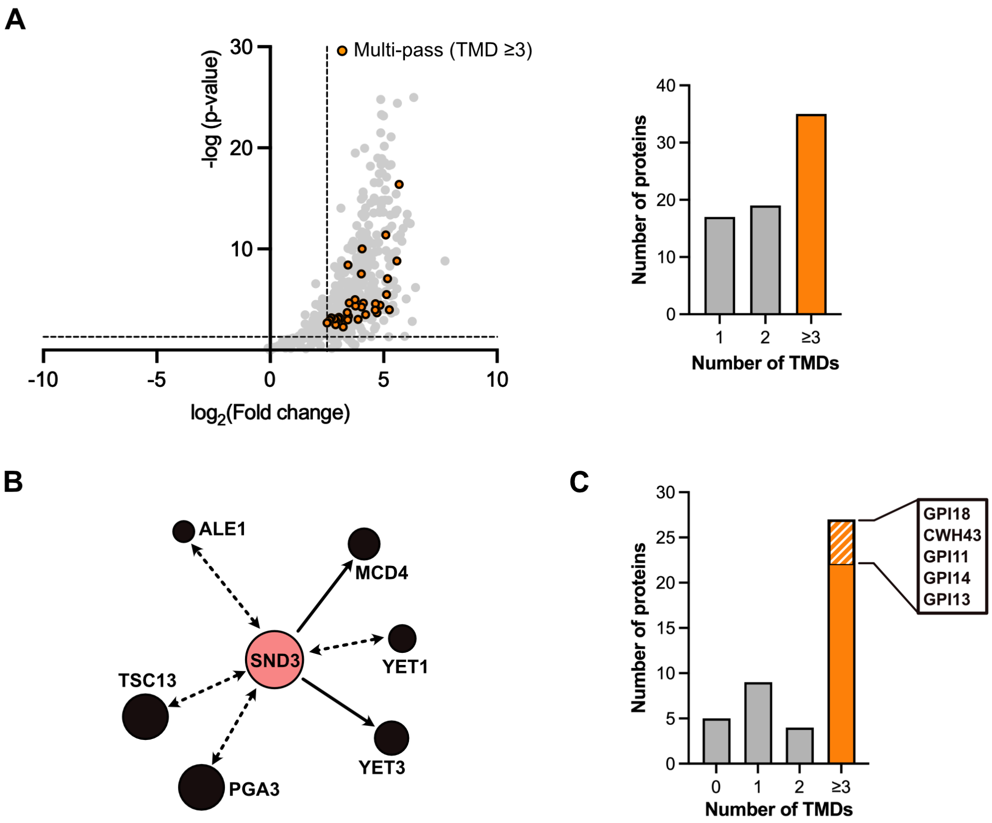

Supplementary Fig. 16. Evidence that the SND3 translocon functions in multipass IMP biogenesis.

(**A**) MS differential abundance analysis showing multipass IMPs are enriched after FLAG-IP from the *C. thermophilum ct*CCDC47-FLAG/*ct*SND3-TwinStrep strain. (Left) Volcano plot showing proteins enriched in samples taken after FLAG-IP over equivalent control samples purified from wild type *C. thermophilum*. Differential abundance analysis was conducted on four independent biological purifications. Multipass IMPs with ≥3 TMDs predicted by DeepTMHMM 1.0[93] were identified and those which are significantly enriched (log_2(fold change) >2.5, p-value <0.05) in our sample are highlighted in orange, excluding components of the SND3 translocon (pairwise comparison, see Methods section). (Right) The absolute number of multipass IMPs (orange) is enriched relative to single or double-spanning IMPs. Source data are provided in the Source Data file. (**B**) The SND3/PHO88 cluster extracted from the yeast-interactome web application (http://www.yeast-interactome.org/)[35] comprises entirely multipass IMPs. Arrows show interactors of SND3, with dashed arrows indicating interactions only supported by profile correlation. (**C**) Proteins whose biogenesis was affected in a *S. cerevisiae Δsbh1/Δsbh2* strain[36]

(SBH1 and SBH2 are the yeast homologues of SEC61β) were classified according to the number of TMDs, as predicted by DeepTMHMM 1.0. Multipass IMPs (orange) are predominantly affected and the subset involved in GPI anchor biosynthesis are highlighted (orange stripes).

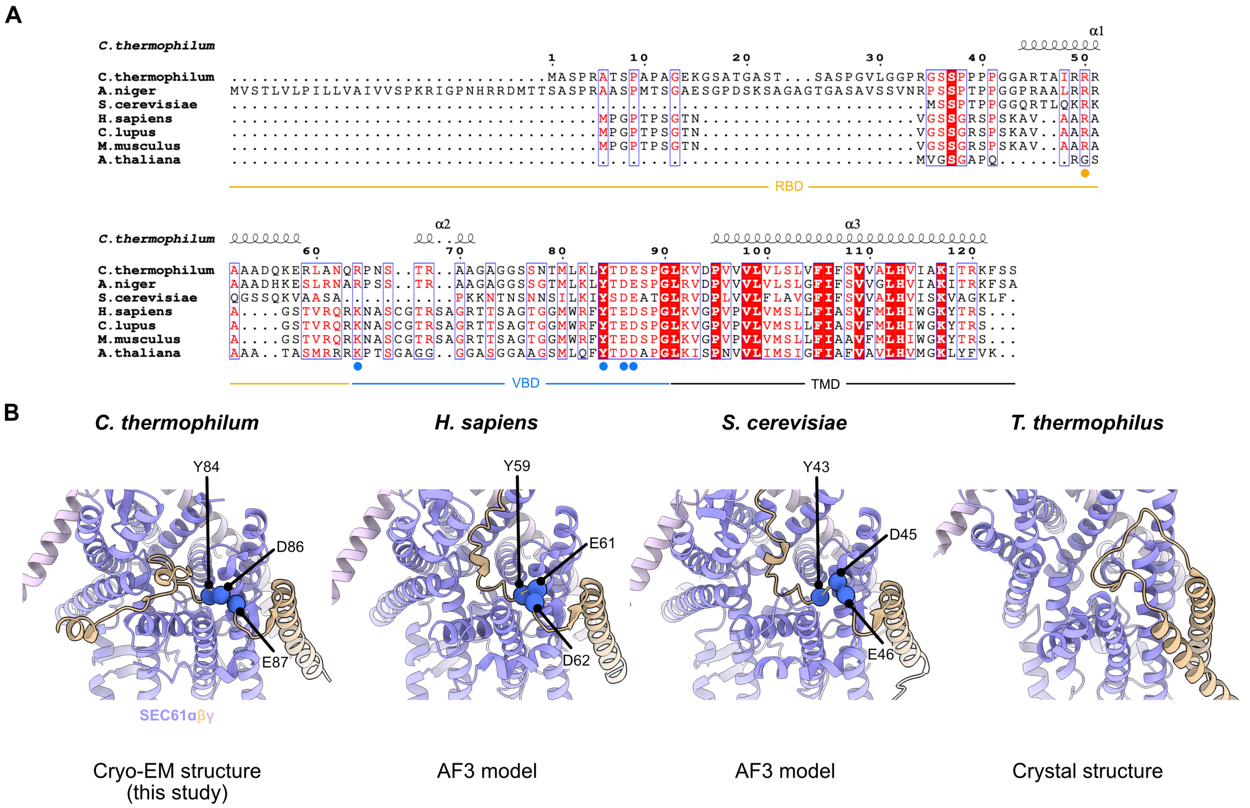

Supplementary Fig. 17. The interaction between the SEC61β vestibule binding domain and SEC61α is conserved.

(**A**) Sequence alignment of SEC61β generated using Clustal Omega[91] and visualised using Espript 3.0[92]. Circles below the alignment highlight conserved residues involved in interactions of the ribosome binding domain (RBD; orange) and vestibule binding domain (VBD; blue) with the ribosome and SEC61α respectively. (**B**) Side-by-side comparison of the *ct*SEC61 translocon structure, the *T. thermophilus* SecYEG crystal structure (PDB 5CH4; RMSD 1.34 Å over 112 atoms) and AF3[21] models for the *H. sapiens* (RMSD 1.03 Å over 367 atoms) and *S. cerevisiae* (RMSD 0.86 Å over 236 atoms) SEC61 translocon after superimposition. In eukaryotes, the analogous interaction between the SEC61β vestibule binding domain and the SEC61α cytosolic vestibule involves three of the highly conserved residues from SEC61β in **A**, shown as blue spheres.

Supplementary Tables

Supplementary Table 1. Primers used for target gene amplification from the *C. thermophilum* genome

Primer	Sequence (5' to 3')
1 kb upstream promoter region + ctSND3 gene	
Forward	CTGCATTATCAGACTCTTTGGCGTATGGGGGCC
Reverse	CTCCTCCTTAACGCCGCCACGGC
343 bp upstream promoter region + ctCCDC47 gene	
Forward	CGACATTGCGCGAGAACAGCGGAATTCGGTTATCAGCATGAACGTGCATG
Reverse	GCAACCGAGGCGGCCGGCCTTAAGCACGGATCGTAGCCT

Supplementary Table 2. System composition (number of molecules) for atomistic MD simulations.

Component	Replicate 1	Replicate 2	Replicate 3
POPC	612	561	561
POPE	432	396	396
POPI	156	143	143
Water	118598	108582	108603
Na^+	459	418	418
Cl^-	327	299	299

Supplementary Table 3. Conditions for equilibrations steps performed before production run during atomistic MD simulations.

	Condition	Integration time step (dt) (fs)	Simulation length (ps)	k (backbone) (kJ mol^{-1} nm^{-2})	k (side chains) (kJ mol^{-1} nm^{-2})	k (lipids) (kJ mol^{-1} nm^{-2})	k (dihedrals) (kJ mol^{-1} rad^{-2})
1	NVT	1	125	4000	2000	1000	1000
2	NVT	1	125	2000	1000	400	400
3	NpT	1	125	1000	500	400	200
4	NpT	2	500	500	200	200	200
5	NpT	2	500	200	50	40	100
6	NpT	2	500	50	0	0	0
7	NpT	2	10,000	0	0	0	0

Supplementary Table 4. Codes used for MD simulation data analysis and visualization.

Figure	Code
3F, 3G	VMD-2.0
S11B	VMD-2.0, distribution analysis: in-house code (Zenodo[89])
S11C	VMD-2.0, density isosurface: VolMap, voltool
S12A and S12B	gmx rms, gmx rmsf
S12C	in-house code (Zenodo[89])
S13A	gmx rmsf
S13B	VMD-2.0
S13C	gmx mindist

Supplementary Table 5. MD simulation checklist for data reproducibility.

**Reliability and reproducibility checklist for molecular dynamics simulations**<br>***All boxes must be marked YES by acceptance unless an N/A option is available**	**Yes**	**N/A**	**Response**<br>**(Please state where this information can be found in the text)**
**1. Convergence of simulations and analysis**			
1a. Is an evaluation presented in the text to show that the property being measured has equilibrated in the simulations (*e.g.* time-course analysis)?	☒		RMSD analysis (Supplementary Fig. 12)
1b. Then, is it described in the text how simulations are split into equilibration and production runs and how much data were analyzed from production runs?	☒		Methods section
1c. Are there at least 3 simulations per simulation condition with statistical analysis?	☒		Methods section
1d. Is evidence provided in the text that the simulation results presented are independent of initial configuration?	☒		Methods section
**2. Connection to experiments**			
2a. Are calculations provided that can connect to experiments (*e.g.* loss or gain in function from mutagenesis, binding assays, NMR chemical shifts, J-couplings, SAXS curves, interaction distances or FRET distances, structure factors, diffusion coefficients, bulk modulus and other mechanical properties, *etc*.)?	☐	N/A	
**3. Method choice**			
3a. Is it described in the text what force field and water model are used and why?	☒		Methods section
3b. Do simulations contain membranes, membrane proteins, intrinsically disordered proteins, glycans, nucleic acids, polymers, or cryptic ligand binding?	☒	☐	Methods section
If 3b is **YES**, are enhanced sampling methods used?	☐	☒	Response not needed if **N/A**
If enhanced sampling methods are used, are the convergence criteria clearly stated?	☐	N/A	
If 3b is **YES**, is it explained in the text why or why not enhanced sampling methods are used?	☐	N/A	
**4. Code and reproducibility**			
4a. Is a table provided describing the system setup, such as simulation box dimensions, total number of atoms, total number of water molecules, salt concentration, lipid composition (number of molecules and type)?	☒		Methods section
4b. Is it described in the text what simulation and analysis software and which versions are used?	☒		Methods section
4c. Are initial coordinate and simulation input files and a coordinate file of the final output provided as supplementary files or in a public repository?	☒		Zenodo, doi: 10.5281/zenodo.16745187
4d. Is there custom code or custom force field parameters?	☒	☐	Methods section
If **YES**, are they provided as supplementary profiles or in a public repository?	☒		Zenodo, doi: 10.5281/zenodo.16745187

Supplementary References

91. Madeira F*, et al.* The EMBL-EBI Job Dispatcher sequence analysis tools framework in 2024. *Nucleic Acids Research* **52**, W521-W525 (2024).

92. Robert X, Gouet P. Deciphering key features in protein structures with the new ENDscript server. *Nucleic Acids Research* **42**, W320-324 (2014).

93. Hallgren J*, et al.* DeepTMHMM predicts alpha and beta transmembrane proteins using deep neural networks. *bioRxiv*, 2022.2004.2008.487609 (2022).
