# Supplementary material for: SND3 is the membrane insertase within a distinct SEC61 translocon complex
Source: Nat Commun. 2025 Oct 29;16:9566. doi: 10.1038/s41467-025-65357-z (PMC12572126; doi:10.1038/s41467-025-65357-z)
Supplement: Supplementary file 2 — Description of Additional Supplementary Files [file 41467_2025_65357_MOESM2_ESM.pdf]

Description of Additional Supplementary Files

File name: Supplementary Movie 1
Description: Structural overview of the ribosome-bound SND3 translocon.
Overview of the cryo-EM structure of the *C. thermophilum* ribosome-bound SND3 translocon. The reconstruction is colored according to the estimated local resolution values (see also Supplementary Fig. 3) and the model of the SND3 translocon is labeled as in Fig. 1C.

File name: Supplementary Movie 2
Description: Lipid scrambling by SND3.
Movie of a coarse-grained (CG) molecular dynamics simulation trajectory showing the scrambling of a lipid molecule (POPC) between the membrane leaflets. SND3 and SEC61a are shown as red and blue surfaces respectively. The transparent yellow spheres represent the PO4 CG beads in the lipid molecules (POPC, POPI, and POPE) and provide reference for the lipid bilayer.
