# Supplementary material for: SND3 is the membrane insertase within a distinct SEC61 translocon complex
Source: Nat Commun. 2025 Oct 29;16:9566. doi: 10.1038/s41467-025-65357-z (PMC12572126; doi:10.1038/s41467-025-65357-z)
Supplement: Supplementary file 5 — Reporting Summary [file 41467_2025_65357_MOESM5_ESM.pdf]

Corresponding author(s): Melanie A. McDowell

Last updated by author(s): Sep 22, 2025

Reporting Summary

Nature Portfolio wishes to improve the reproducibility of the work that we publish. This form provides structure for consistency and transparency in reporting. For further information on Nature Portfolio policies, see our Editorial Policies and the Editorial Policy Checklist.

Statistics

For all statistical analyses, confirm that the following items are present in the figure legend, table legend, main text, or Methods section.

n/a	Confirmed	
☐	☒	The exact sample size (*n*) for each experimental group/condition, given as a discrete number and unit of measurement
☐	☒	A statement on whether measurements were taken from distinct samples or whether the same sample was measured repeatedly
☐	☒	The statistical test(s) used AND whether they are one- or two-sided<br>*Only common tests should be described solely by name; describe more complex techniques in the Methods section.*
☐	☒	A description of all covariates tested
☐	☒	A description of any assumptions or corrections, such as tests of normality and adjustment for multiple comparisons
☐	☒	A full description of the statistical parameters including central tendency (e.g. means) or other basic estimates (e.g. regression coefficient) AND variation (e.g. standard deviation) or associated estimates of uncertainty (e.g. confidence intervals)
☐	☒	For null hypothesis testing, the test statistic (e.g. *F*, *t*, *r*) with confidence intervals, effect sizes, degrees of freedom and *P* value noted<br>*Give P values as exact values whenever suitable.*
☒	☐	For Bayesian analysis, information on the choice of priors and Markov chain Monte Carlo settings
☒	☐	For hierarchical and complex designs, identification of the appropriate level for tests and full reporting of outcomes
☒	☐	Estimates of effect sizes (e.g. Cohen's *d*, Pearson's *r*), indicating how they were calculated

Our web collection on statistics for biologists contains articles on many of the points above.

Software and code

Policy information about availability of computer code

Data collection	Negative stain EM images were acquired with Digital Micrograph (Gatan) and Cryo-EM images were acquired using EPU 3.8 (Thermo Scientific).
Data analysis	Cryo-EM images were processed with Relion 4.0, Relion 5.0, cryoSPARC v.4.4.0 and MotionCorr2. Molecular models were built using AlphaFold 3.0, Coot 0.9.8, UCSF-ChimeraX 1.7.1 and Model Angelo then refined using Phenix 1.21, Coot and ISOLDE. Local resolution analysis was performed using ResMap. Structures were assessed by MolProbity and visualised with UCSF-ChimeraX. Sequence analysis was performed with Clustal Omega and visualised with ESPript 3.0. Transmembrane domains were identified using DeepTMHMM 1.0 and sequence homologues were identified using OrthoDB v12.0. MS data analysis was performed using FragPipe v21.1, MSFragger 4.0, DIA-NN and the MS-DAP script (v1.2.1). Atomistic molecular dynamics simulations were performed using the CHARMM-GUI server, the PPM 2.0 server, the CHARMM36m force field, the CHARMM-modified TIP3P model, the GROMACS-2022.4 simulation package and LINCS and SETTLE algorithms. Coarse-grained simulations were performed using the martinize2 script, the insane script and the GROMACS-2022.4 package. VMD-2.0, VolMap, gmx rms/rmsf/mindist and in-house code (https://doi.org/10.5281/zenodo.16745187) were used to visualise and analyse MD simulation data.

For manuscripts utilizing custom algorithms or software that are central to the research but not yet described in published literature, software must be made available to editors and reviewers. We strongly encourage code deposition in a community repository (e.g. GitHub). See the Nature Portfolio guidelines for submitting code & software for further information.

Data

Policy information about availability of data

All manuscripts must include a data availability statement. This statement should provide the following information, where applicable:

- Accession codes, unique identifiers, or web links for publicly available datasets
- A description of any restrictions on data availability
- For clinical datasets or third party data, please ensure that the statement adheres to our policy

Coordinates for the Chaetomium thermophilum SND3 translocon bound to the 60S ribosomal subunit have been deposited in the Protein Data Bank under accession number 9I78 [https://doi.org/10.2210/pdb9I78/pdb]. The respective cryo-EM map has been deposited in the Electron Microscopy Data Bank under accession code EMD-52656 [https://www.ebi.ac.uk/emdb/EMD-52656], and the cryo-EM map with improved density for the TRAPα luminal domain under accession code EMD-52829 [https://www.ebi.ac.uk/emdb/EMD-52829]. The MS proteomics data have been deposited to the ProteomeXchange Consortium via the PRIDE partner repository88 with the dataset identifier PXD060914 [http://proteomecentral.proteomexchange.org/cgi/GetDataset?ID=PXD060914]. The MD simulation files are available on Zenodo as entry 16745187 [https://doi.org/10.5281/zenodo.16745187]. Source data are provided with this paper.

Research involving human participants, their data, or biological material

Policy information about studies with human participants or human data. See also policy information about sex, gender (identity/presentation), and sexual orientation and race, ethnicity and racism.

Reporting on sex and gender	Our study does not involve human research participants.
Reporting on race, ethnicity, or other socially relevant groupings	Our study does not involve human research participants.
Population characteristics	Our study does not involve human research participants.
Recruitment	Our study does not involve human research participants.
Ethics oversight	Our study does not involve human research participants.

Note that full information on the approval of the study protocol must also be provided in the manuscript.

Field-specific reporting

Please select the one below that is the best fit for your research. If you are not sure, read the appropriate sections before making your selection.

☒ Life sciences ☐ Behavioural & social sciences ☐ Ecological, evolutionary & environmental sciences

For a reference copy of the document with all sections, see nature.com/documents/nr-reporting-summary-flat.pdf

Life sciences study design

All studies must disclose on these points even when the disclosure is negative.

Sample size	For the cryo-EM sample, at least 8 grids were pre-screened to identify the optimal grid for data collection. 14039 movies were collected and the final map was generated from 119,533 particles. The complexes illustrated in Supplementary Fig. S1 could be generated from at least two independent purifications. Differential abundance analysis was performed from four independent biological replicates and atomistic MD simulations were performed three times. No formal sample size calculations were performed, rather replicate numbers were chosen to be sure of reproducibility and particles contributing to the final cryo-EM map were selected as a result of the processing pipeline in Supplementary Fig. 2.
Data exclusions	For cryo-EM, poor grids were discarded, only micrographs with CTF_fit_resolution < 4 Å were used for processing and only particles corresponding to the ribosome-bound SND3 translocon were selected during the classification stages of data processing.<br>For MS analysis, all database searches were restricted to tryptic peptides with a length of 7-30 amino acids, up to two missed cleavages and with a minimum of one unique peptide per protein group. Carbamidomethylation of Cysteine was set as a fixed modification and oxidation of Methionine as well as N-terminal acetylation were set as variable modifications. Percolator was used to estimate the number of false positive identifications and the results were filtered for a false discovery rate (FDR) < 0.01.<br>For differential abundance analysis, pairwise comparisons between control and ctSND3-TwinStrep/CCDC47-FLAG samples were performed using MSqRob with qvalue thresholds of 0.01 and a minimum of 1 unique peptide detected in at least 2 of the 4 independent biological replicates per condition. Those with log2(fold change) >2.5, p-value <0.05 are highlighted as significantly enriched in the volcano plot and considered for further analysis. For the analysis in Supplementary Fig. 16A, components of the SND3 translocon are excluded.
Replication	One cryo-EM dataset was collected providing >750,000 particles for subsequent processing. We since collected a second dataset from the FLAG-IP sample, yielding a very similar map.<br>For differential abundance MS analysis of solution SND3 translocon samples, four independent purifications were analysed for both control and ctSND3-TwinStrep/CCDC47-FLAG containing membranes. Three independent atomistic MD simulations were performed and used to

derive the plots in Supplementary Figs. 11B, 13A and 13C. The replicates for Supplementary Fig. 12B are shown in the Source Data. Purifications (as shown by the gels/blots in Supplementary Fig. 1A) were performed at least twice, always leading to the purification of the same complexes. All findings were reproducible.

Randomization	For cryo-EM, particles were randomly distributed in the ice and during processing were randomly divided into two groups for refinement and resolution estimation.
Blinding	For cryo-EM, MS and MD simulations, blinding is not relevant as the variables are not subjective or influenced by the experimenter.

Reporting for specific materials, systems and methods

We require information from authors about some types of materials, experimental systems and methods used in many studies. Here, indicate whether each material, system or method listed is relevant to your study. If you are not sure if a list item applies to your research, read the appropriate section before selecting a response.

Materials & experimental systems

n/a	Involved in the study
☐	☒ Antibodies
☒	☐ Eukaryotic cell lines
☒	☐ Palaeontology and archaeology
☒	☐ Animals and other organisms
☒	☐ Clinical data
☒	☐ Dual use research of concern
☒	☐ Plants

Methods

n/a	Involved in the study
☒	☐ ChIP-seq
☒	☐ Flow cytometry
☒	☐ MRI-based neuroimaging

Antibodies

Antibodies used	monoclonal anti-FLAG M2 antibody produced in mouse (Sigma-Aldrich, F3165-1MG) Lot: SLCJ3741, 1:1000 dilution<br>monoclonal anti-Strep antibody produced in mouse (IBA, 2-1507-001) Lot: 1507-0054, 1:2500 dilution<br>Peroxidase AffiniPure Goat Anti-Mouse IgG (H+L) (JacksonImmuno Research, 115-035-146) 1:5000 dilution
Validation	For anti-FLAG M2, numerous citations on manufacturer website: https://www.sigmaaldrich.com/DE/en/search/f3165?focus=papers&page=1&perpage=30&sort=relevance&term=F3165&type=citation_search<br>For anti-Strep antibody, numerous citations on manufacturer website: https://www.iba-lifesciences.com/detail/9c7864c598eb4dd2abeef8a55b5beebb

Plants

Seed stocks	*Report on the source of all seed stocks or other plant material used. If applicable, state the seed stock centre and catalogue number. If plant specimens were collected from the field, describe the collection location, date and sampling procedures.*
Novel plant genotypes	*Describe the methods by which all novel plant genotypes were produced. This includes those generated by transgenic approaches, gene editing, chemical/radiation-based mutagenesis and hybridization. For transgenic lines, describe the transformation method, the number of independent lines analyzed and the generation upon which experiments were performed. For gene-edited lines, describe the editor used, the endogenous sequence targeted for editing, the targeting guide RNA sequence (if applicable) and how the editor was applied.*
Authentication	*Describe any authentication procedures for each seed stock used or novel genotype generated. Describe any experiments used to assess the effect of a mutation and, where applicable, how potential secondary effects (e.g. second site T-DNA insertions, mosiacism, off-target gene editing) were examined.*
